# Absolute and Relative Morbidity Burdens Attributable to Various Illnesses and Injuries Among Active Component Members of the U.S. Coast Guard, 2024

**Published:** 2025-09-20

The U.S. Coast Guard is a military service that operates under the authority of the U.S. Department of Homeland Security, providing law and maritime safety enforcement, marine and environmental protection and military naval support. ^1 , 2^ It is the second smallest service of the U.S. Armed Forces, with approximately 45,940 active component service members, and the only military service operating outside the authority of the Department of Defense (DOD). Coast Guard personnel are eligible to use DOD health care facilities, but because many service members are not stationed near a DOD installation, the Coast Guard operates primary care clinics in areas with sufficiently large Coast Guard populations, which is limited to providing primary care. ^1^

Recent research indicates that Coast Guard beneficiaries (i.e., active duty service members, reservists entitled to specific care, retirees, dependents) face challenges obtaining care meeting access standards due to several factors, including staffing shortages at Coast Guard clinics, data gaps, a lack of information to ensure member assignments optimally address the health needs of dependents, and more. ^1^ A higher proportion of civilian hospitalizations among Coast Guard members has been noted ^3^ ; this difference may extend to ambulatory care as well. The *MSMR* annual morbidity burden report excluded hospitalization data for the U.S. Coast Guard service members from 2016 through 2021 due to missing data. ^3 , 4^

To quantify the impacts of various illnesses and injuries among members of the active component of the U.S. Coast Guard in 2024, this summary report employs the same disease classification system and morbidity burden measures that were used in the general active component burden analysis.

## Methods

The population for this analysis included all individuals who served in the active component of the Coast Guard at any time during the surveillance period of January 1, 2024 through November 30, 2024. The methodology for summarizing absolute and relative Coast Guard morbidity burdens in 2024 is identical to the methodology described on page 5 of this issue that determined the absolute and relative burdens attributed to various illnesses and injuries among the active component of the U.S. Armed Forces.

## Results

In 2024, a total of 36,686 Coast Guard service members had 470,239 total medical encounters, which included 10,143 hospital bed days reported, for a rate of 0.28 hospital bed days per Coast Guard member who experienced at least 1 medical encounter, either ambulatory or hospitalization.

### Morbidity burden, by category

In 2024, more active component Coast Guard members experienced medical encounters for injury (n=16,297) than any other morbidity-related category **(Figure 1a)** . Second-most frequent in terms of hospital bed days, injury accounted for over one-fifth (22.1%) of all medical encounters **(Figure 1b)** .

**FIGURE 1a.**
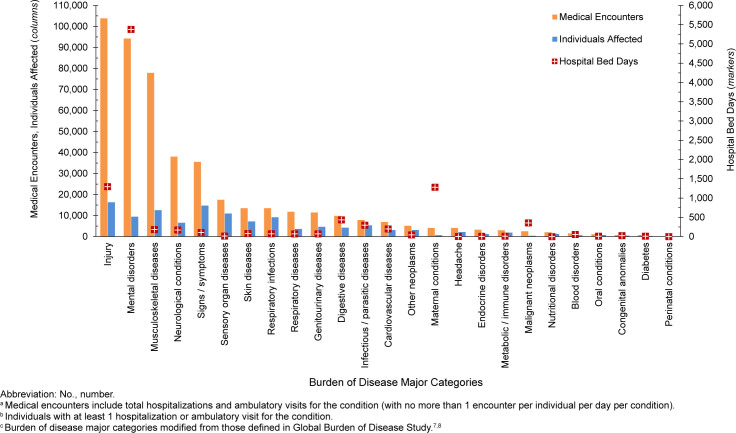
Numbers of Medical Encounters ^a^ , Individuals Affected ^b^ and Hospital Bed Days by Burden of Disease Major Category ^c^ , Active Component, U.S. Coast Guard, 2024

**FIGURE 1b.**
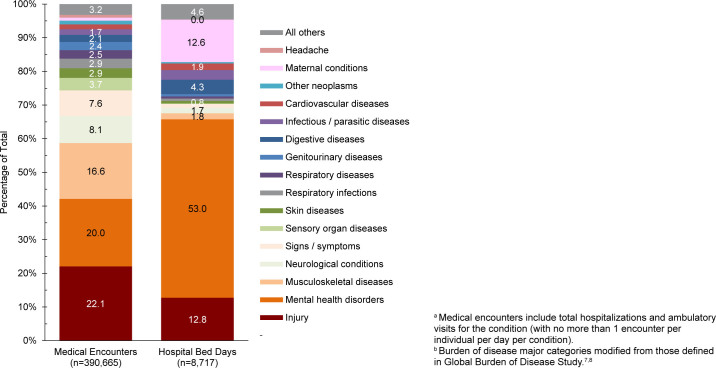
Percentage of Medical Encounters ^a^ and Hospital Bed Days Attributable to Burden of Disease Major Categories ^b^ , Active Component, U.S. Coast Guard, 2024

Mental health disorders accounted for more hospital bed days (n=5,376) than any other morbidity-related category, constituting over half (53.0%) of all hospital bed days, and fifth in terms of numbers of individuals affected **(Figures 1a** , **1b)** . Combined, injury and mental health disorders accounted for over three-fifths (65.8%) of all hospital bed days and more than two-fifths (42.1%) of all medical encounters.

**What are the new findings?**

In 2024, injuries, mental health disorders, and musculoskeletal diseases were the categories of medical conditions associated with the most medical encounters, greatest numbers of members affected, and largest numbers of hospital days among active duty Coast Guard members, similar to Department of Defense active component service members. When compared to 2023, medical encounters rose by 6.2%, hospital bed days increased by 13.7%, and major category conditions increased by 5.7%. In 2024, COVID-19 accounted for 0.3% of total medical encounters, a decrease from 0.4% in 2023, and 0.2% of hospital bed days reported in 2024.

**What is the impact on readiness and force health protection?**

The major condition categories in this report present health challenges for members of the U.S. Coast Guard and affect their service readiness. Loss of duty availability related to illness and injury diminishes Coast Guard personnel readiness. Coast Guard members have unique occupational exposures that may benefit from specific risk reduction programs to mitigate these threats.

Maternal conditions (pregnancy complications, delivery), accounted for a relatively large proportion of all hospital bed days (n=1,280, 12.6%) but a much smaller proportion of total medical encounters (n=4,094 0.9%) **(Figures 1a** , **1b)** . Maternal conditions were the most prevalent medical condition among female active component Coast Guard members. Women comprised approximately one-sixth (16.4%) of the active duty Coast Guard in 2024.

### Medical encounters, by condition

**FIGURE 2.**
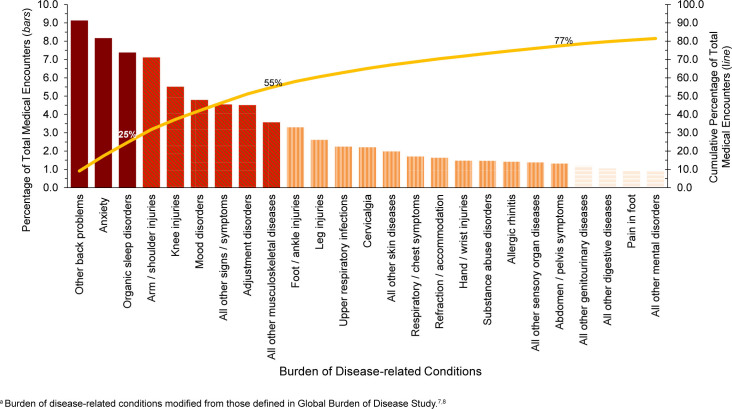
Percentage and Cumulative Percentage Distribution, Burden of Disease-related Conditions ^a^ that Accounted for the Most Medical Encounters, Active Component, U.S. Coast Guard, 2024

In 2024, 5 disease-related conditions accounted for more than one-third (37.3%) of all illness- and injury-related medical encounters among active component Coast Guard members: other back problems (includes lower back pain, other dorsalgia), anxiety disorders, organic sleep disorders (e.g., obstructive sleep apnea, insomnia), arm / shoulder injuries, and knee injuries **(Figure 2)** . Moreover, the 10 conditions associated with the most medical encounters constituted more than half (58.0%) of all illness- and injury-related medical encounters.

The disease-related conditions in 2024 that predominantly accounted for medical encounters among active component Coast Guard members were injuries, mental health disorders, and musculoskeletal diseases. Among the reported injuries, arm / shoulder (7.1%), knee (5.5%), foot / ankle (3.3%), and leg (2.6%) injuries accounted for the most medical encounters **(Figure 2** and **Table)** . Anxiety (8.2%), mood (4.8%), adjustment (4.5%), and substance abuse disorders (1.5%) were the 4 most frequent mental health disorder diagnoses. Other back problems (9.1%), all other musculoskeletal diseases (3.6%), and cervicalgia (2.2%) constituted the most medical encounters among musculoskeletal disorders. COVID-19 accounted for 0.3% of total medical encounters in 2024.

**TABLE:** Health Care Burdens Attributable to Various Diseases and Injuries, Active Component, U.S. Coast Guard, 2024 ^a^

Major Category Condition ^b^	Medical Encounters ^c^		Individuals Affected ^d^		Hospital Bed Days	
	No.	Rank ^e^	No.	Rank ^e^	No.	Rank ^e^
Total	470,239				10,143	
**Injury, poisoning**	**103,865**				**1,296**	
Arm, shoulder injury	33,448	4	4,987	9	203	11
Knee injury	25,919	5	4,048	12	44	31
Foot, ankle injury	15,502	10	3,939	13	85	19
Leg injury	12,275	11	2,461	20	233	9
Hand, wrist injury	6,928	17	2,616	17	15	48
Head, neck injury	2,962	29	1,607	27	191	13
Back, abdomen injury	2,325	33	1,090	39	129	16
Unspecified injury	1,130	53	821	46	3	81
Environmental injury / poisoning	1,090	56	840	45	1	90
Other complications, not otherwise specified	988	57	541	57	326	5
Other harm from external causes	812	60	595	54	19	43
Poisoning, non-drug	270	97	177	88	4	75
All other injuries	108	117	91	102	0	93
Poisoning, drugs	55	127	26	124	43	32
Other burns	42	132	37	114	0	93
Other superficial injury	11	142	8	137	0	93
**Mental disorders**	**94,235**				**5,376**	
Anxiety disorder	38,411	2	5,517	6	806	4
Mood disorder	22,535	6	2,607	18	2,001	2
Adjustment disorder	21,211	8	3,636	14	279	7
Substance abuse disorder	6,888	18	534	59	2,106	1
All other mental disorders	4,151	25	1,124	38	12	53
Psychotic disorder	505	77	44	112	164	14
Personality disorder	196	108	35	116	4	75
Somatoform disorder	184	113	70	105	4	75
Tobacco dependence	154	115	101	99	0	93
**Musculoskeletal diseases**	**77,914**				**181**	
Other back problems	42,939	1	7,087	3	119	18
All other musculoskeletal diseases	16,782	9	5,259	8	50	28
Cervicalgia	10,376	13	1,960	23	3	81
Pain in foot	4,195	24	1,719	24	0	93
Osteoarthritis	2,086	36	961	41	2	85
Other shoulder disorders	715	63	302	73	0	93
Other knee disorders	636	72	240	82	7	62
Rheumatoid arthritis	185	112	70	105	0	93
**Signs, symptoms, ill-defined conditions**	**35,565**				**109**	
All other signs and symptoms	21,371	7	10,332	1	74	21
Respiratory, chest signs and symptoms	8,005	15	4,905	10	13	50
Abdomen, pelvis signs and symptoms	6,189	21	3,565	15	22	39
**Neurological conditions**	**38,052**				**171**	
Organic sleep disorder	34,714	3	5,869	5	4	75
All other neurological conditions	1,578	44	580	56	155	15
Chronic pain	883	58	375	65	0	93
Other mononeuritis, upper / lower limbs	454	80	208	85	0	93
Epilepsy	222	104	69	107	7	62
Multiple sclerosis	159	114	28	123	5	72
Parkinson's disease	42	132	2	144	0	93
**Sensory organ diseases**	**17,475**				**13**	
Refraction, accommodation	7,678	16	6,601	4	0	93
All other sensory organ diseases	6,492	20	4,205	11	13	50
Hearing disorders	2,551	31	1,649	25	0	93
Glaucoma	675	69	421	61	0	93
Cataracts	79	120	55	108	0	93
**Infectious and parasitic diseases**	**7,874**				**294**	
All other infectious and parasitic diseases	3,832	27	2,418	21	254	8
COVID-19	1,307	48	1,167	36	20	41
Tinea skin infection	1,186	51	895	43	0	93
Unspecified viral infection	691	66	647	52	6	64
Diarrheal disease	416	83	359	68	8	59
Sexually transmitted disease (STD)	340	90	266	79	0	93
Chlamydia	57	126	52	110	0	93
Intestinal nematode infection	22	138	14	131	0	93
Tuberculosis	7	143	5	139	0	93
Bacterial meningitis	6	144	1	149	6	64
Hepatitis B, C	6	144	5	139	0	93
Malaria	2	147	2	144	0	93
Tropical cluster	2	147	2	144	0	93
**Skin diseases**	**13,533**				**77**	
All other skin diseases	9,313	14	5,322	7	71	22
Sebaceous gland disease	2,539	32	1,523	31	0	93
Contact dermatitis	1,681	41	1,315	33	6	64
**Respiratory diseases**	**11,822**				**68**	
Allergic rhinitis	6,660	19	1,431	32	0	93
All other respiratory diseases	1,748	39	1,032	40	67	23
Chronic sinusitis	1,317	47	807	47	0	93
Deviated nasal septum	694	65	389	64	1	90
Asthma	606	75	312	72	0	93
Chronic obstructive pulmonary disease	447	81	365	67	0	93
Chronic rhinitis	350	88	242	81	0	93
**Genitourinary diseases**	**11,418**				**68**	
All other genitourinary diseases	5,616	22	2,781	16	19	43
Female genital pain	1,378	45	629	53	0	93
Menstrual disorder	1,139	52	689	49	0	93
UTI, cystitis	809	61	586	55	6	64
Other breast disorders	788	62	406	63	3	81
Kidney stones	679	68	277	76	24	37
Nephritis, nephrosis	424	82	109	98	16	45
Vaginitis, vulvitis	348	89	281	75	0	93
Benign prostatic hypertrophy	237	101	137	93	0	93
**Digestive diseases**	**9,880**				**432**	
All other digestive diseases	4,952	23	2,482	19	298	6
Esophagus disease	2,151	35	1,259	34	11	55
Other gastroenteritis, colitis	1,632	43	868	44	64	25
Constipation	479	79	356	69	1	90
Inguinal hernia	365	87	144	91	0	93
Appendicitis	187	111	77	103	52	27
Cirrhosis of liver	63	125	10	135	0	93
Peptic ulcer disease	51	130	29	122	6	64
**Respiratory infections**	**13,500**				**73**	
Upper respiratory infection	10,551	12	7,830	2	25	36
Lower respiratory infection	1,679	42	1,242	35	48	29
Otitis media	1,270	49	958	42	0	93
**Cardiovascular diseases**	**6,914**				**196**	
All other cardiovascular diseases	3,458	28	1,587	28	128	17
Essential hypertension	2,831	30	1,527	30	2	85
Ischemic heart disease	296	94	129	95	11	55
Cerebrovascular disease	215	105	99	100	45	30
Inflammatory disease	80	119	39	113	4	75
Rheumatic heart disease	34	135	32	120	6	64
**Other neoplasms**	**5,117**				**43**	
Benign skin neoplasm	1,993	38	1,628	26	0	93
All other neoplasms	1,728	40	1,151	37	24	37
Neoplasm of uncertain behavior of skin	831	59	670	51	0	93
Lipoma	369	86	233	84	3	81
Uterine leiomyoma	196	108	95	101	16	45
**Headache**	**4,084**				**2**	
Headache	4,084	26	2,178	22	2	85
**Maternal conditions**	**4,094**				**1,280**	
Pregnancy complications	2,186	34	518	60	835	3
All other maternal disorders	1,196	50	353	70	193	12
Delivery	399	84	270	77	232	10
Ectopic pregnancy, miscarriage, abortion	225	103	75	104	5	72
Puerperium complications	88	118	54	109	15	48
**Metabolic and immune disorders**	**3,184**				**11**	
Unspecified disorder of pituitary gland	194	110	110	97	0	93
Lipoid metabolism disorder	2,086	36	1,540	29	0	93
Other metabolic disorders	513	76	243	80	11	55
Gout	312	92	165	89	0	93
Immune disorder	79	120	34	117	0	93
**Endocrine disorders**	**3,064**				**6**	
Testicular hypofunction	1,098	55	369	66	0	93
Hypothyroidism	673	70	337	71	0	93
Other thyroid disorders	662	71	283	74	2	85
All other endocrine disorders	383	85	190	86	4	75
Polycystic ovarian syndrome	248	100	122	96	0	93
**Malignant neoplasms**	**2,501**				**354**	
Melanoma, other skin cancers	501	78	182	87	41	33
Lymphoma, multiple myeloma	311	93	30	121	76	20
Leukemia	286	95	22	126	65	24
Colon, rectal cancers	278	96	18	128	40	34
All other malignant neoplasms	267	99	45	111	59	26
Breast cancer	237	101	17	129	2	85
Testicular cancer	198	106	36	115	9	58
Brain cancer	132	116	11	133	40	34
Trachea, bronchus, lung cancers	71	122	4	141	6	64
Mouth, oropharynx cancers	69	123	4	141	16	45
Prostate cancer	55	127	6	138	0	93
Thyroid cancer	48	131	10	135	0	93
Cervix uteri cancer	24	137	11	133	0	93
Bladder cancer	21	139	2	144	0	93
Corpus uteri cancer	1	151	1	149	0	93
Pancreatic cancer	1	151	1	149	0	93
Stomach cancer	1	151	1	149	0	93
**Nutritional disorders**	**2,009**				**0**	
Overweight, obesity	1,375	46	761	48	0	93
All other nutritional disorders	632	73	538	58	0	93
Protein-energy malnutrition	2	147	2	144	0	93
**Blood disorders**	**1,516**				**53**	
All other blood disorders	680	67	270	77	21	40
Iron deficiency anemia	336	91	130	94	12	53
Other non-deficiency anemias	269	98	156	90	20	41
Hereditary anemia	197	107	140	92	0	93
Other deficiency anemias	34	135	26	124	0	93
**Diabetes mellitus**	**709**				**8**	
Diabetes mellitus	709	64	237	83	8	59
**Oral conditions**	**1,157**				**8**	
All other oral conditions	1,104	54	671	50	8	59
Periodontal disease	35	134	33	119	0	93
Dental caries	18	140	17	129	0	93
**Congenital anomalies**	**735**				**24**	
All other congenital anomalies	618	74	417	62	5	72
Congenital heart disease	64	124	34	117	6	64
Other circulatory anomalies	53	129	20	127	13	50
** Conditions arising during perinatal period ^f^**	**22**				**0**	
All other perinatal anomalies	17	141	12	132	0	93
Birth asphyxia, birth trauma	3	146	3	143	0	93
Low birth weight	2	147	1	149	0	93

Abbreviations: No., number; NOS, not otherwise specified; UTI, urinary tract infection; STD, sexually transmitted disease.

a Burden of disease major categories and burden of disease-related conditions modified from those defined in Global Burden of Disease Study. ^7 , 8^

b Medical encounters include total hospitalizations and ambulatory visits for the condition (with no more than 1 encounter per individual per day per condition).

c Individuals with at least 1 hospitalization or ambulatory visit for the condition.

d Rank is based on number of encounters, individuals affected, or hospital bed days in the respective columns within the listing of 157 burden-related disease conditions. Nine pairs of tied values for medical encounters and 11 pairs of tied values for individuals affected were given the same ranking. For hospital bed days, there were 61 conditions with the rank of 93 (0); 17 other conditions had tied rankings.

e Conditions affecting newborns erroneously coded on service member medical records.

### Individuals affected, by condition

The 10 categories of conditions that affected the most Coast Guard members in 2024 were all other signs and symptoms, upper respiratory infections, other back problems, refraction / accommodation, organic sleep disorders, anxiety, all other skin diseases, all other musculoskeletal diseases, arm and shoulder conditions, and respiratory and chest issues. COVID-19 affected 1,167 Coast Guard members, ranking thirty-sixth for number of individuals affected, a slight drop from thirty-third in 2023.

### Hospital bed days, by condition

**FIGURE 3.**
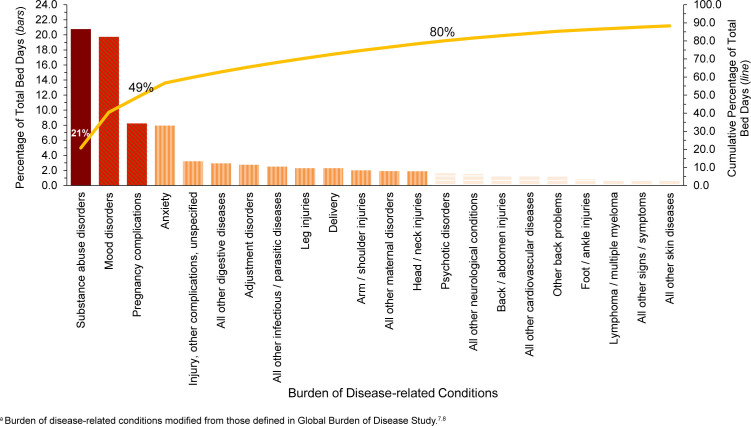
Percentage and Cumulative Percentage Distribution, Burden of Disease-related Conditions ^a^ that Accounted for the Most Hospital Bed Days, Active Component, U.S. Coast Guard, 2024

In 2024, substance abuse and mood disorders accounted for about two-fifths (40.5%) of all hospital bed days **(Figure 3)** . Four mental health disorders (substance abuse, mood, anxiety, adjustment) and 2 maternal conditions (pregnancy complications, delivery) combined accounted for more than three-fifths (61.7%) of all hospital bed days **(Table** and **Figure 3)** . About 12.8% of all hospital bed days were attributable to injuries. In 2024, 0.2% hospitalizations of active component Coast Guard members were due to COVID-19 **(Table)** .

## Discussion

Health care use within the Coast Guard was similar to the DOD when measured by total encounters and persons affected in 2024. The Coast Guard rate was 12.8 encounters per person (470,239 per 36,686 individuals), compared to the DOD rate of 12.3 encounters per person (14,197,058 per 1,150,913 individuals). The Coast Guard had a lower rate of hospitalization, however, with only 0.28 bed days per individual; the DOD reported 0.33 bed days per individual (378,693 per 1,150,913 individuals).

Compared to 2023, the number of Coast Guard medical encounters and hospital bed days increased by 6.2% and 13.7%, respectively, and the major category conditions increased by 5.7%. While the number of Coast Guard medical encounters and the number of major category conditions increased in 2024, the rate of change was significantly lower than in 2023 (13.3% and 12.7%, respectively). Conversely, the rate of change in hospital bed days in 2024 (13.7%) was significantly higher than in 2023 (2.3%). Mental health disorders resulted in more hospital stays than any other morbidity-related category, and mental health-related medical encounters increased by 15.5% compared to last year. In 2024, the number of individuals affected decreased by 2.0% compared to 2023.

This report is consistent with the major findings of prior annual reports on morbidity burdens among active component U.S. service members. Injuries, mental health disorders, and musculoskeletal diseases were the categories of medical conditions associated with the most medical encounters, largest numbers of affected service members, and greatest numbers of hospital bed days; maternal conditions accounted for the most hospital bed days, followed by mental health disorders. When examining ICD codes to the fourth digit character, Coast Guard and DOD service members shared many disease-related conditions: other back problems within the musculoskeletal disease major diagnostic category; arm / shoulder and knee injuries within the injury major diagnostic category; anxiety disorders in the mental health disorder major diagnostic category; and organic sleep disorders within the neurological condition major diagnostic category.

COVID-19 did not account significantly for medical encounters in 2024 compared to 2023: COVID accounted for 0.2% of hospital bed days in 2024, compared to none (0) in 2023. In addition to the waning of the pandemic, active component service members represent a relatively young and healthy population that is less likely to experience severe consequences from COVID-19 infection.

Preventable illnesses and injuries, which contribute disproportionately to morbidity and health care burdens, should be high priority targets for intervention, research, and resources. In a 2018 survey, Coast Guard members reported several mental health issues including serious psychological distress, failure to receive mental health services despite need, and other preventable risky health behaviors. ^5^ Reliable access to health care is crucial for ensuring service members remain healthy and prepared for their missions. A lack of data hinders the Coast Guard from fully understanding healthcare accessibility issues, however. ^1^ To accurately portray the true burden of disease in this population, addressing and resolving the data gaps resulting from Coast Guard hospitalizations to civilian facilities is critical and should be a priority. Improving data collection processes and systems is crucial to addressing barriers to accessing health care.

Providing a matrix of major diseases each year enables the identification, in comparison with previous reports, of potentially avoidable health conditions among military personnel, and their proximate causes. Morbidity burden report findings can aid prioritization of effective interventions, provision of necessary care, and evaluation of their impacts and cost-effectiveness. ^6^
